# Seroprevalence of Zika Virus in Wild African Green Monkeys and Baboons

**DOI:** 10.1128/mSphere.00392-16

**Published:** 2017-03-08

**Authors:** Connor R. Buechler, Adam L. Bailey, Andrea M. Weiler, Gabrielle L. Barry, Meghan E. Breitbach, Laurel M. Stewart, Anna J. Jasinska, Nelson B. Freimer, Cristian Apetrei, Jane E. Phillips-Conroy, Clifford J. Jolly, Jeffrey Rogers, Thomas C. Friedrich, David H. O’Connor

**Affiliations:** a Department of Pathology, University of Wisconsin—Madison, Madison, Wisconsin, USA; b Wisconsin National Primate Research Center, Madison, Wisconsin, USA; c Center for Neurobehavioral Genetics, Semel Institute for Neuroscience and Human Behavior, University of California, Los Angeles, California, USA; d Center for Vaccine Research, University of Pittsburgh, Pittsburgh, Pennsylvania, USA; e Department of Microbiology and Molecular Genetics, University of Pittsburgh, Pittsburgh, Pennsylvania, USA; f Department of Neuroscience, Washington University School of Medicine, and Department of Anthropology, Washington University, Saint Louis, Missouri, USA; g Department of Anthropology, New York University, New York, New York, USA; h Human Genome Sequencing Center, Baylor College of Medicine, Houston, Texas, USA; UT Southwestern Medical Center

**Keywords:** false-positive reactions, *Flavivirus*, sensitivity and specificity, seroepidemiologic studies, Zika virus, Zika virus infection

## Abstract

Zika virus (ZIKV) has recently spread through the Americas and has been associated with a range of health effects, including birth defects in children born to women infected during pregnancy. Although the natural reservoir of ZIKV remains poorly defined, the virus was first identified in a captive “sentinel” macaque monkey in Africa in 1947. However, the virus has not been reported in humans or nonhuman primates (NHPs) in Africa outside Gabon in over a decade. Here, we examine ZIKV infection in 239 wild baboons and African green monkeys from South Africa, the Gambia, Tanzania, and Zambia using combinations of unbiased deep sequencing, quantitative reverse transcription-PCR (qRT-PCR), and an antibody capture assay that we optimized using serum collected from captive macaque monkeys exposed to ZIKV, dengue virus, and yellow fever virus. While we did not find evidence of active ZIKV infection in wild NHPs in Africa, we found variable ZIKV seropositivity of up to 16% in some of the NHP populations sampled. We anticipate that these results and the methodology described within will help in continued efforts to determine the prevalence, natural reservoir, and transmission dynamics of ZIKV in Africa and elsewhere.

**IMPORTANCE** Zika virus (ZIKV) is a mosquito-borne virus originally discovered in a captive monkey living in the Zika Forest of Uganda, Africa, in 1947. Recently, an outbreak in South America has shown that ZIKV infection can cause myriad health effects, including birth defects in the children of women infected during pregnancy. Here, we sought to investigate ZIKV infection in wild African primates to better understand its emergence and spread, looking for evidence of active or prior infection. Our results suggest that up to 16% of some populations of nonhuman primate were, at some point, exposed to ZIKV. We anticipate that this study will be useful for future studies that examine the spread of infections from wild animals to humans in general and those studying ZIKV in primates in particular.

**Podcast**: A podcast concerning this article is available.

## Abstract (precis)

Zika virus (ZIKV) is a mosquito-borne virus originally discovered in a captive monkey living in the Zika Forest of Uganda, Africa, in 1947. Recently, an outbreak in South America has shown that ZIKV infection can cause myriad health effects, including birth defects in the children of women infected during pregnancy. Here, we sought to investigate ZIKV infection in wild African primates to better understand its emergence and spread, looking for evidence of active or prior infection. Our results suggest that up to 16% of some populations of nonhuman primate were, at some point, exposed to ZIKV. We anticipate that this study will be useful for future studies that examine the spread of infections from wild animals to humans in general and those studying ZIKV in primates in particular.

## INTRODUCTION

Zika virus (ZIKV) is a positive-sense single-stranded RNA virus (family *Flaviviridae*, genus *Flavivirus*) that recently emerged to cause widespread human infection in Micronesia, French Polynesia, Singapore, and the Americas. The cause of ZIKV’s recent global spread is poorly understood, and its natural reservoir remains poorly defined. ZIKV was first isolated from a captive rhesus macaque (*Macaca mulatta*) in Uganda in 1947 (1). Since then, ZIKV has been isolated from humans, monkeys, and mosquitoes living in Africa, Southeast Asia, Micronesia, French Polynesia, and the Americas (2). Monkeys in Africa often live in close association with humans and are likely involved in the zoonotic transmission of flaviviruses, including ZIKV, mediated by mosquito vectors (3). While previous serosurveys showed the presence of ZIKV in both humans and nonhuman primates (NHPs) in Africa in the 1950s through 1980s, these surveys have not since been updated, and ZIKV has not been reported in monkeys or humans in Africa outside Gabon in over a decade (2, 3). In addition, past serosurveys were prone to significant cross-reactivity with other flaviviruses, such as dengue virus (DENV), West Nile virus (WNV), and yellow fever virus (YFV), complicating their interpretation (2–7). Here we sought to better understand ZIKV prevalence in wild African primates using unbiased deep sequencing, ZIKV-specific quantitative reverse transcription-PCR (qRT-PCR), and a ZIKV-specific antibody capture assay, assessing for antibody cross-reactivity to other flaviviruses using serum from experimentally infected macaque monkeys.

## RESULTS

To investigate the prevalence of ZIKV and anti-ZIKV antibodies in wild African NHPs, we obtained blood from four groups of NHPs: 14 Chacma-Kinda hybrid baboons (*Papio kindae* × *Papio ursinus griseipes*) from Kafue National Park in Zambia, 41 yellow baboons (*Papio cynocephalus*) from Mikumi National Park in Tanzania, 25 African green monkeys (AGMs) from the Gambia (*Chlorocebus sabaeus*), and 159 AGMs sampled from multiple sites across South Africa (*Chlorocebus pygerythrus*; see reference 8 for a detailed map of sampling sites).

To assess the prevalence of active ZIKV infection, we performed unbiased deep sequencing on a subset of NHP plasma samples described above. Sequencing did not identify ZIKV reads in any of the samples investigated, although other RNA viruses, including lentiviruses, pegiviruses, and arteriviruses, were detected (8–14).

**FIG 1:**
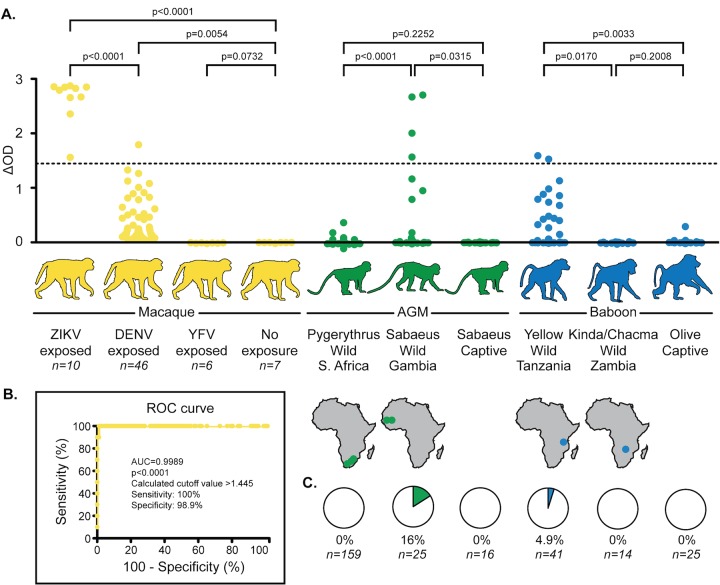
ZIKV ELISA results by NHP type and prior exposure. (A) Reactivity of the anti-ZIKV antibody capture ELISA to serum/plasma collected from macaques (yellow), AGMs (green), and baboons (blue). Anti-ZIKV antibody titers were compared among different groups within each NHP genus using a two-tailed unpaired *t* test. For macaques, these data were also used to generate the receiver operating characteristic (ROC) curve. The geographic region from which samples were collected from wild NHPs is indicated in the maps under each column. (B) ROC curve, plotting the true-positive rate (TPR; sensitivity) against the false-positive rate (FPR; 1 specificity) for potential discrimination thresholds. Units are presented here as percentages rather than fractions. Potential discrimination thresholds were found by sorting all of the values from captive macaques and averaging adjacent values. The area under the curve (AUC) indicates the ability of the test to discriminate between primates with ZIKV and those without, with a perfect test having an AUC of 1 and a test with no ability to discriminate having an AUC of 0.5. Both AUC and the significance compared to a test with an AUC of 0.5 were computed using GraphPad Prism (GraphPad Software, Inc., La Jolla CA). The potential discrimination value that maximized the likelihood ratio (TPR/FPR) was 1.445, and this value was adopted as the empirical positivity threshold, depicted by the dashed line in panel A. (C) Data points above the dashed line in panel A were deemed “positive” and used to estimate the prevalence of ZIKV exposure in each population.

We also assessed the prevalence of prior ZIKV infection by antibody capture enzyme-linked immunosorbent assay (ELISA). Compared to captive ZIKV-naive baboons, several wild baboons from Tanzania had significantly elevated levels of anti-ZIKV antibodies (Fig. 1). In contrast, baboons from Zambia had no evidence of prior ZIKV exposure. Similarly, several AGMs from The Gambia displayed evidence of prior ZIKV exposure while those from South Africa had anti-ZIKV antibody levels comparable to those of captive ZIKV-naive AGMs. None of the samples containing anti-ZIKV antibodies contained ZIKV RNA as measured by highly sensitive quantitative RT-PCR (qRT-PCR).

A well-described problem in assessing ZIKV seroprevalence is the cross-reactivity of antibodies to other flaviviruses with ZIKV antibody capture assays (2–7). To assess whether the anti-ZIKV antibodies we detected in Tanzanian baboons and Gambian AGMs were elicited by genuine ZIKV infection, we sought to rigorously characterize the cross-reactivity of our ZIKV antibody capture assay. We tested sera from captive macaques infected with DENV serotypes 1 to 4 as well as animals challenged with DENV serotypes 1 to 4 after tetravalent vaccination to simulate repeated heterologous exposure to flaviviruses closely related to ZIKV and found that these sera registered with our ZIKV ELISA at levels above the manufacturer-recommended positivity threshold of a change in optical density (ΔOD) of >0.3 (Fig. 1; see Table S1 in the supplemental material). However, sera from macaques vaccinated with YFV registered at levels comparable to uninfected animals. To control for cross-reactivity to DENV and reduce the assay’s false-positive rate, we generated a receiver operating characteristic (ROC) curve and determined a positivity threshold based on empirical data from captive macaques (Fig. 1B). The area under this curve, 0.9989, indicated that the assay could consistently distinguish between samples from ZIKV-exposed and ZIKV-naive animals, even those repeatedly and recently exposed to DENV. The optimal positivity threshold, ΔOD of >1.445, showed a specificity of 98.9% and a sensitivity of 100%. To validate our sample set at this threshold, we blindly tested sera from ZIKV-exposed and ZIKV-naive animals and were able to accurately determine their exposure history (see Fig. S1 in the supplemental material). At this threshold, 4 of 25 (16%) Gambian AGMs showed evidence of prior exposure to ZIKV, while 2 of 41 (4.9%) Tanzanian baboons tested positive for anti-ZIKV antibody (Fig. 1C; see Table S2 in the supplemental material).

10.1128/mSphere.00392-16.5 TABLE S1  ELISA data from captive animals with known clinical histories. The superscript “a” indicates that samples were prepared as described in Materials and Methods. ZIKV (FP) is virus derived from a French Polynesian isolate (Zika virus/*H. sapiens*-tc/FRA/2013/FrenchPolynesia-01_v1c1). ZIKV (MR766) is the ZIKV type strain first discovered in Uganda in 1947. Download TABLE S1, PDF file, 1.6 MB. Copyright © 2017 Buechler et al. 2017 Buechler et al. This content is distributed under the terms of the Creative Commons Attribution 4.0 International license.

10.1128/mSphere.00392-16.1 FIG S1  Blind test of ZIKV ELISA after setting a new positivity criterion. Download FIG S1, PDF file, 0.04 MB. Copyright © 2017 Buechler et al. 2017 Buechler et al. This content is distributed under the terms of the Creative Commons Attribution 4.0 International license.

10.1128/mSphere.00392-16.2 FIG S2  ELISA values from DENV-exposed and DENV-vaccinated and -challenged macaques. Download FIG S2, PDF file, 0.1 MB. Copyright © 2017 Buechler et al. 2017 Buechler et al. This content is distributed under the terms of the Creative Commons Attribution 4.0 International license.

10.1128/mSphere.00392-16.3 FIG S3  ELISA values from DENV-exposed rhesus macaques separated by serotype. Download FIG S3, PDF file, 0.1 MB. Copyright © 2017 Buechler et al. 2017 Buechler et al. This content is distributed under the terms of the Creative Commons Attribution 4.0 International license.

10.1128/mSphere.00392-16.4 FIG S4  ELISA values from DENV-vaccinated and -challenged rhesus macaques separated by serotype. Download FIG S4, PDF file, 0.1 MB. Copyright © 2017 Buechler et al. 2017 Buechler et al. This content is distributed under the terms of the Creative Commons Attribution 4.0 International license.

10.1128/mSphere.00392-16.6 TABLE S2  Positive ELISA results from wild African primates. The superscript “a” indicates that samples were prepared as described in Materials and Methods. Download TABLE S2, PDF file, 0.1 MB. Copyright © 2017 Buechler et al. 2017 Buechler et al. This content is distributed under the terms of the Creative Commons Attribution 4.0 International license.

## DISCUSSION

We assessed the prevalence of ZIKV exposure in four wild primate populations in Africa: baboons (genus *Papio*) from Tanzania and Zambia and AGMs (genus *Chlorocebus*) from South Africa and the Gambia. We saw no evidence of active infection, but our results suggest prior ZIKV exposure in Tanzanian baboons and Gambian AGMs. As ZIKV causes an acute viral infection in nonpregnant NHPs and humans, the lack of active ZIKV infection in this cross-sectional study is not entirely surprising. Additionally, our use of qRT-PCR and unbiased deep sequencing enabled us to rule out the presence of low-titer ZIKV infection as well as the presence of other flaviviruses circulating in these NHP populations at the time of sampling. Nevertheless, these methods do not rule out the possibility of low-copy-number infection with other flaviviruses, although such courses are not typical of ZIKV or closely related flaviviruses.

We observed a range of anti-ZIKV antibody titers in wild NHPs. Several factors could account for this wide range in antibody titers, including waning immunity from a ZIKV infection in the distant past, infection with a flavivirus that elicits an antibody response that cross-reacts with our ZIKV antibody capture assay (e.g., DENV), or repeated flaviviral infections that might increase the avidity of pan-flaviviral antibodies. While we assessed cross-reactivity in this assay using samples from animals previously exposed to either DENV or YFV, two flaviviruses known to cross-react in ZIKV antibody capture assays, it should be noted that there are many other flaviviruses present in nature with the potential to elicit antibodies that may cross-react with this assay. Nonetheless, the empirically derived positivity threshold for our anti-ZIKV ELISA, based on samples from animals infected with ZIKV or closely related flaviviruses, and our validation of this method suggest that the antibodies we detected in wild NHPs were elicited by genuine ZIKV infection. The ZIKV seroprevalence estimates reported here are well below many of those noted in previous studies (2), possibly reflecting the lower false-positive rate of this method and providing a more accurate estimate of the incidence of ZIKV infection in the NHP communities surveyed.

**FIG 2:**
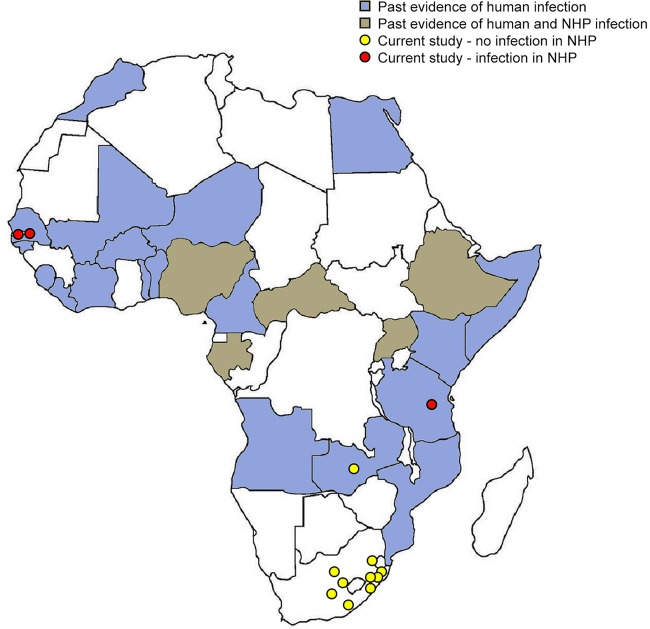
Geographic representation of ZIKV detection in wild African primates. Countries where human ZIKV infection has been previously detected are shown in blue, countries where human and nonhuman primate ZIKV infection have both been previously detected are shown in brown, locations in which nonhuman primate ZIKV infection was detected in the present study are shown with red dots, and locations in which nonhuman primate ZIKV infection was not detected in the present study are shown with yellow dots. A review of previous serosurvey studies can be found in reference 2.

We saw more ZIKV-exposed animals in NHPs from the northern areas of sub-Saharan Africa, even given the more extensive sampling performed on similar species in southern Africa (Fig. 2). These results suggest that the distribution of ZIKV infection may be determined more by environmental or geographical factors, such as the prevalence of particular mosquito vectors or tropical forest conditions, than host species, although further work is needed to substantiate this hypothesis. We also saw evidence of ZIKV infection in areas of Africa where ZIKV has not been reported in humans or primates in over a decade (2, 3), potentially pointing to a persistent and previously unrecognized reservoir. Ultimately, we believe that the methods reported here will be useful for larger serosurveys of wild animals to more accurately determine the prevalence of ZIKV infection. Such studies will be vital for further understanding the natural reservoir and transmission dynamics of ZIKV in Africa and elsewhere.

## MATERIALS AND METHODS

### Sample collection.

Four populations of wild nonhuman primates were included in this study—African green monkeys (AGMs) from across South Africa (*Chlorocebus pygerythrus*) and the Gambia (*Chlorocebus sabaeus*) and baboons from Kafue National Park in Zambia (Kinda × Chacma hybrid baboons [*Papio kindae* × *Papio ursinus griseipes*]) and Mikumi National Park in Tanzania (yellow baboons [*Papio cynocephalus*]). All research involving nonhuman primates was conducted according to regulations set forth by the Animal Welfare Act and approved by the appropriate wildlife authorities and institutional animal care and use committees. Briefly, all animals were sedated prior to blood collection and were released back to their social group without incident following sample collection and recovery from anesthesia. After initial collection, samples were stored on dry ice, in liquid nitrogen, or otherwise below −40°C. Sampling of AGMs was approved by the Interfaculty Animal Ethics Committee (project no. 13/2010) at the University of the Free State and by the University of Wisconsin—Milwaukee Animal Care and Use Committee (protocol 07-08 32) using methods described previously (15). Sampling of yellow baboons in Tanzania was performed using standard methods for field studies of baboons as described previously (16). The field work in Tanzania was reviewed and approved by The Tanzanian National Scientific Research Council, the Serengeti Wildlife Institute, Tanzanian National Parks, Washington University, and Yale University, with additional approval through United States NSF grant BNS83-03506. Sampling of hybrid Kinda × grayfooted-Chacma baboons in Zambia was performed in compliance with the rules of the Zambian Wildlife Authority and the rules of the animal care and use committees from Baylor College of Medicine (AN-5538), Washington University School of Medicine (protocol 20120269), New York University (protocol 10-1349), and applicable national laws. Samples from Tanzania were collected in 1985 and 1986; samples from the Gambia, South Africa, and Zambia were collected between 2010 and 2014.

### Deep sequencing.

Samples from South Africa, Zambia, and Tanzania were processed for deep sequencing as described previously (13). Briefly, viral RNA was isolated from approximately 200 μl of plasma, and cDNA synthesis was primed using random hexamer primers. Samples were fragmented, and sequencing adaptors were added using a Nextera DNA sample preparation kit (Illumina, San Diego, CA). Deep sequencing was performed on an Illumina MiSeq. Sequence data were analyzed using CLC Genomics Workbench (version 6.5) software (CLC Bio, Aarhus, Denmark) and Geneious R9 software (Biomatters, Auckland, New Zealand). Low-quality reads (Phred quality score of <Q30) and short reads (<100 bp) were removed. Samples were then compared against all viral sequences in the NCBI GenBank database as of 22 June 2016 (17) using BLAST.

### ELISA.

All samples were tested for ZIKV-specific immunoglobulin G (IgG) against recombinant ZIKV nonstructural protein 1 (NS1) using ELISA kits designed by XPressBio (Frederick, MD). Samples were diluted 1:50, and the assay was performed according to the manufacturer’s instructions, with all tests performed in duplicate. Briefly, diluted samples were applied to wells precoated with ZIKV NS1 protein or a negative-control antigen. After incubation and washing with a Tris buffer, a secondary anti-simian antibody conjugated to horseradish peroxidase was added. This substrate was visualized after another incubation and wash step with the addition of ABTS [2,2′-azinobis(3-ethylbenzthiazolinesulfonic acid)] peroxidase substrate. Test values were determined by subtracting the optical density (OD) at 405 nm of the well precoated with negative-control antigen from that of the well precoated with ZIKV NS1, resulting in a ΔOD value for each test. In addition to the samples from wild African primates, groups of baboons (*n* = 25) and AGMs (*n* = 16) were also tested after being raised in captivity with no known flaviviral exposure. Rhesus macaques (*Macaca mulatta*) were tested after being infected with the African strain of ZIKV (ZIKV-MR766; *n* = 3) or the French-Polynesian/South American strain of ZIKV (ZIKV-FP; *n* = 7), vaccinated for YFV (*n* = 6), or infected with DENV without prior vaccination (DENV-1 [*n* = 6], DENV-2 [*n* = 6], DENV-3 [*n* = 12], DENV-4 [*n* = 6]). A group of cynomolgus macaques (*Macaca fascicularis*) was tested after DENV-1 (*n* = 4), DENV-2 (*n* = 4), DENV-3 (*n* = 4), or DENV-4 (*n* = 4) challenge following administration of a tetravalent DENV vaccine, simulating repeated heterologous exposure to multiple flaviviruses closely related to ZIKV. Four of the unexposed and four of the ZIKV-FP-infected rhesus macaques were tested separately in a blind confirmation of the experimentally determined positivity criterion. Exposure dates ranged from 2 years to 2 months prior to sample collection for both experimentally infected and vaccinated animals.

### qRT-PCR.

Seven samples from the Gambia and one from South Africa that tested above the manufacturer-recommended ELISA threshold of an ΔOD of >0.3 were also subjected to highly sensitive qRT-PCR to discern whether viral RNA was still present. Similar samples from Tanzania were not included due to sample availability. No samples from Zambia tested above the manufacturer-recommended positivity threshold. The qRT-PCR was performed as described previously (18), using the SuperScript III Platinum one-step qRT-PCR system (Invitrogen, Carlsbad, CA) on the LightCycler 480 instrument (Roche Diagnostics, Indianapolis, IN), with forward primer CGYTGCCCAACACAAGG, reverse primer CCACYAAYGTTCTTTTGCABACAT, and probe AGCCTACCTTGAYAAGCARTCAGACACYCAA (19). The virus concentration was determined by interpolation onto an internal standard curve composed of seven 10-fold serial dilutions of a synthetic ZIKV RNA fragment based on ZIKV strain H/PF/2013.
