# Supplementary material for: Seroprevalence of Zika Virus in Wild African Green Monkeys and Baboons
Source: mSphere. 2017 Mar 8;2(2):e00392-16. doi: 10.1128/mSphere.00392-16 (PMC5343173; doi:10.1128/mSphere.00392-16)
Supplement: TABLE S1 [file sph002172248st1.pdf]

| Non-human primate | Prior exposure | ΔOD[a] | Assay result |
|---|---|---|---|
| *Macaca mulatta* | ZIKV (MR766) | 2.356 | + |
| *Macaca mulatta* | ZIKV (MR766) | 1.56 | + |
| *Macaca mulatta* | ZIKV (MR766) | 2.658 | + |
| *Macaca mulatta* | ZIKV (FP) | 2.669 | + |
| *Macaca mulatta* | ZIKV (FP) | 2.873 | + |
| *Macaca mulatta* | ZIKV (FP) | 2.847 | + |
| *Macaca mulatta* | ZIKV (FP) | 2.851 | + |
| *Macaca mulatta* | ZIKV (FP) | 2.824 | + |
| *Macaca mulatta* | ZIKV (FP) | 2.855 | + |
| *Macaca mulatta* | ZIKV (FP) | 2.793 | + |
| *Macaca mulatta* | DENV-1 | 0.219 | - |
| *Macaca mulatta* | DENV-1 | 1.125 | - |
| *Macaca mulatta* | DENV-1 | 1.329 | - |
| *Macaca mulatta* | DENV-1 | 0.444 | - |
| *Macaca mulatta* | DENV-1 | 0.783 | - |
| *Macaca mulatta* | DENV-1 | 1.265 | - |
| *Macaca mulatta* | DENV-2 | 0.063 | - |
| *Macaca mulatta* | DENV-2 | 0.159 | - |
| *Macaca mulatta* | DENV-2 | 0.263 | - |
| *Macaca mulatta* | DENV-2 | 0.145 | - |
| *Macaca mulatta* | DENV-2 | 0.28 | - |
| *Macaca mulatta* | DENV-2 | 0.06 | - |
| *Macaca mulatta* | DENV-3 | 0.073 | - |
| *Macaca mulatta* | DENV-3 | 0.079 | - |
| *Macaca mulatta* | DENV-3 | 0.125 | - |
| *Macaca mulatta* | DENV-3 | 0.132 | - |
| *Macaca mulatta* | DENV-3 | 0.181 | - |
| *Macaca mulatta* | DENV-3 | 0.219 | - |
| *Macaca mulatta* | DENV-3 | 0.235 | - |
| *Macaca mulatta* | DENV-3 | 0.247 | - |
| *Macaca mulatta* | DENV-3 | 0.315 | - |
| *Macaca mulatta* | DENV-3 | 0.42 | - |
| *Macaca mulatta* | DENV-3 | 0.557 | - |
| *Macaca mulatta* | DENV-3 | 0.645 | - |
| *Macaca mulatta* | DENV-4 | 0.196 | - |
| *Macaca mulatta* | DENV-4 | 1.081 | - |
| *Macaca mulatta* | DENV-4 | 0.101 | - |
| *Macaca mulatta* | DENV-4 | 0.812 | - |
| *Macaca mulatta* | DENV-4 | 1.008 | - |
| *Macaca mulatta* | DENV-4 | 0.157 | - |
| *Macaca mulatta* | YFV vax | -0.016 | - |
| *Macaca mulatta* | YFV vax | -0.015 | - |
| *Macaca mulatta* | YFV vax | -0.011 | - |
| *Macaca mulatta* | YFV vax | -0.009 | - |
| *Macaca mulatta* | YFV vax | -0.004 | - |
| *Macaca mulatta* | YFV vax | -0.009 | - |
| *Macaca fascicularis* | Dengue vaccination (tetravalent) and subsequent DENV-1 challenge | 0.11 | - |
| *Macaca fascicularis* | Dengue vaccination (tetravalent) and subsequent DENV-1 challenge | 0.149 | - |
| *Macaca fascicularis* | Dengue vaccination (tetravalent) and subsequent DENV-1 challenge | 0.46 | - |
| *Macaca fascicularis* | Dengue vaccination (tetravalent) and subsequent DENV-1 challenge | 1.791 | + |
| *Macaca fascicularis* | Dengue vaccination (tetravalent) and subsequent DENV-2 challenge | 0.224 | - |
| *Macaca fascicularis* | Dengue vaccination (tetravalent) and subsequent DENV-2 challenge | 0.614 | - |
| *Macaca fascicularis* | Dengue vaccination (tetravalent) and subsequent DENV-2 challenge | 0.894 | - |
| *Macaca fascicularis* | Dengue vaccination (tetravalent) and subsequent DENV-2 challenge | 0.912 | - |
| *Macaca fascicularis* | Dengue vaccination (tetravalent) and subsequent DENV-3 challenge | 0.043 | - |
| *Macaca fascicularis* | Dengue vaccination (tetravalent) and subsequent DENV-3 challenge | 0.148 | - |
| *Macaca fascicularis* | Dengue vaccination (tetravalent) and subsequent DENV-3 challenge | 0.463 | - |
| *Macaca fascicularis* | Dengue vaccination (tetravalent) and subsequent DENV-3 challenge | 0.828 | - |
| *Macaca fascicularis* | Dengue vaccination (tetravalent) and subsequent DENV-4 challenge | 0.045 | - |
| *Macaca fascicularis* | Dengue vaccination (tetravalent) and subsequent DENV-4 challenge | 0.219 | - |
| *Macaca fascicularis* | Dengue vaccination (tetravalent) and subsequent DENV-4 challenge | 0.501 | - |
| *Macaca fascicularis* | Dengue vaccination (tetravalent) and subsequent DENV-4 challenge | 0.526 | - |
| *Macaca mulatta* | none | -0.006 | - |
| *Macaca mulatta* | none | -0.003 | - |
| *Macaca mulatta* | none | -0.003 | - |
| *Macaca mulatta* | none | -0.004 | - |
| *Macaca mulatta* | none | -0.003 | - |
| *Macaca mulatta* | none | -0.002 | - |
| *Macaca mulatta* | none | -0.02 | - |
| *Papio anubis* | none | -0.002 | - |
| *Papio anubis* | none | -0.002 | - |
| *Papio anubis* | none | 0.034 | - |
| *Papio anubis* | none | -0.001 | - |
| *Papio anubis* | none | -0.001 | - |
| *Papio anubis* | none | 0.006 | - |
| *Papio anubis* | none | -0.001 | - |
| *Papio anubis* | none | 0.002 | - |
| *Papio anubis* | none | 0.011 | - |
| *Papio anubis* | none | 0.002 | - |
| *Papio anubis* | none | -0.009 | - |
| *Papio anubis* | none | -0.009 | - |
| *Papio anubis* | none | -0.002 | - |
| *Papio anubis* | none | 0 | - |
| *Papio anubis* | none | 0.003 | - |
| *Papio anubis* | none | 0.01 | - |
| *Papio anubis* | none | 0.289 | - |
| *Papio anubis* | none | -0.01 | - |
| *Papio anubis* | none | 0.003 | - |
| *Papio anubis* | none | 0.036 | - |
| *Papio anubis* | none | -0.002 | - |
| *Papio anubis* | none | -0.023 | - |
| *Papio anubis* | none | 0.006 | - |
| *Papio anubis* | none | -0.008 | - |
| *Papio anubis* | none | -0.007 | - |
| *Chlorocebus sabaeus* | none | -0.012 | - |
| *Chlorocebus sabaeus* | none | -0.009 | - |
| *Chlorocebus sabaeus* | none | -0.006 | - |
| *Chlorocebus sabaeus* | none | -0.003 | - |
| *Chlorocebus sabaeus* | none | -0.002 | - |
| *Chlorocebus sabaeus* | none | 0.004 | - |
| *Chlorocebus sabaeus* | none | -0.006 | - |
| *Chlorocebus sabaeus* | none | -0.005 | - |
| *Chlorocebus sabaeus* | none | -0.002 | - |
| *Chlorocebus sabaeus* | none | -0.01 | - |
| *Chlorocebus sabaeus* | none | -0.017 | - |
| *Chlorocebus sabaeus* | none | -0.006 | - |
| *Chlorocebus sabaeus* | none | -0.008 | - |
| *Chlorocebus sabaeus* | none | 0.008 | - |
| *Chlorocebus sabaeus* | none | -0.002 | - |
| *Chlorocebus sabaeus* | none | 0.004 | - |
