# Supplementary figures and images for: Seroprevalence of Zika Virus in Wild African Green Monkeys and Baboons
Source: mSphere. 2017 Mar 8;2(2):e00392-16. doi: 10.1128/mSphere.00392-16 (PMC5343173; doi:10.1128/mSphere.00392-16)

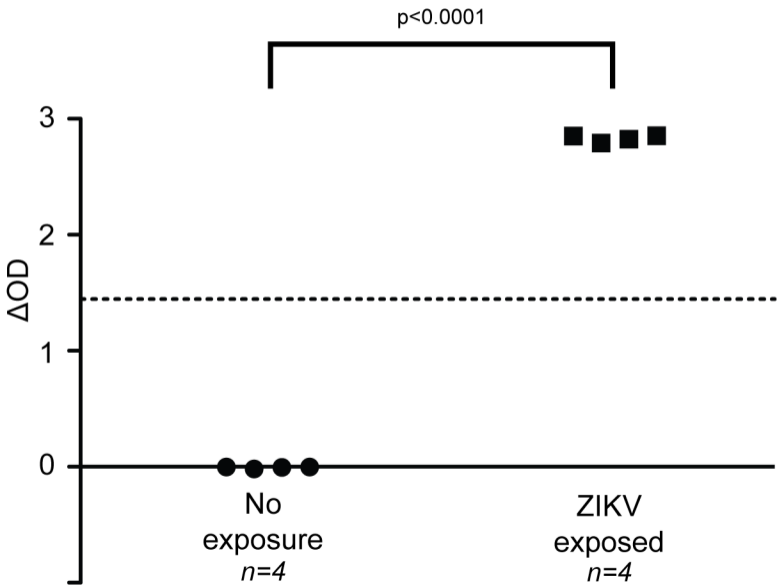

Supplement: FIG S1 [file sph002172248sf3.pdf]

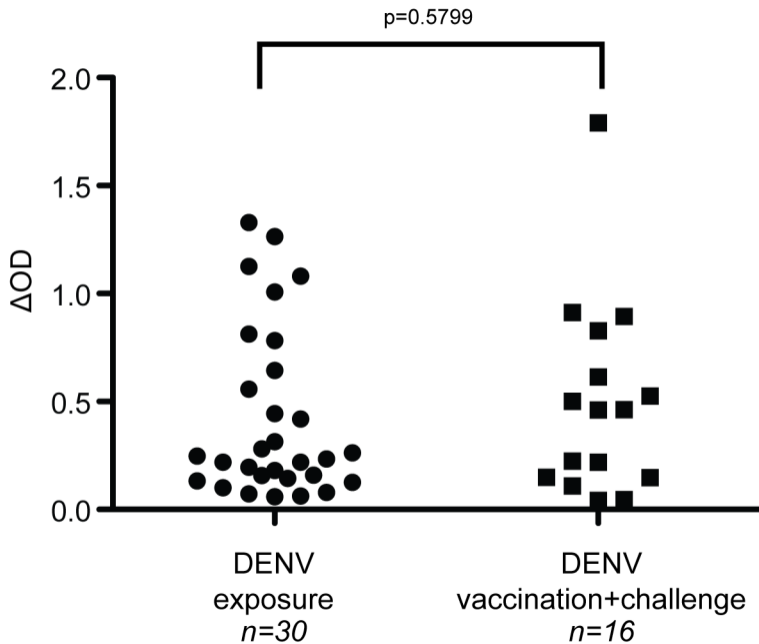

Supplement: FIG S2 [file sph002172248sf4.pdf]

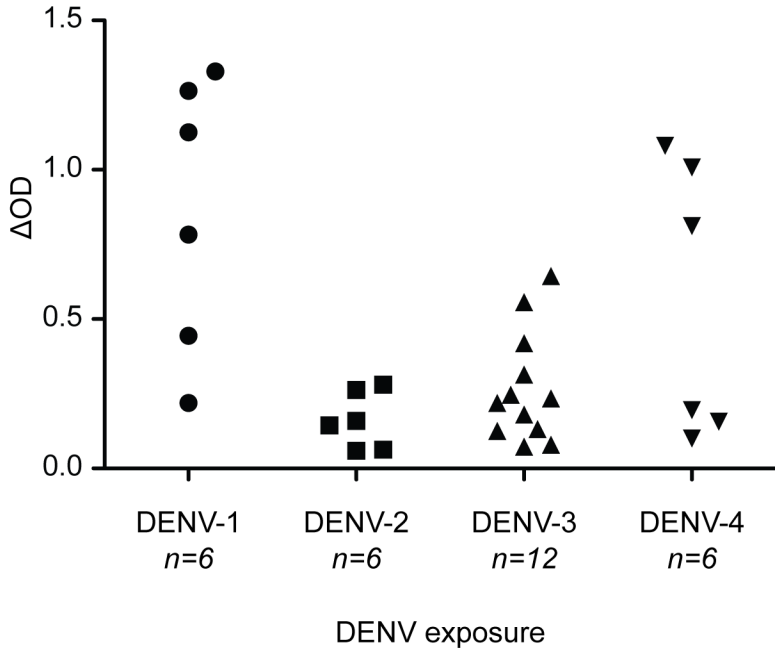

Supplement: FIG S3 [file sph002172248sf5.pdf]

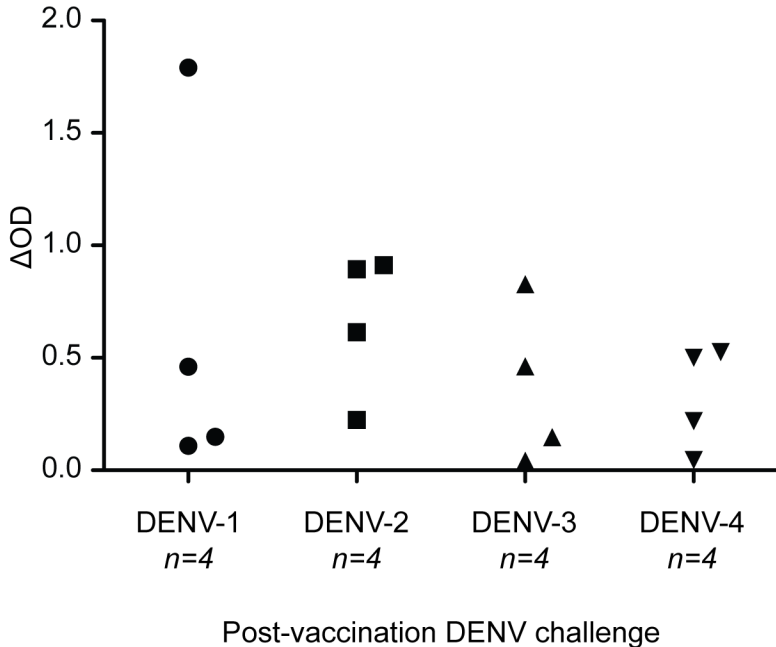

Supplement: FIG S4 [file sph002172248sf6.pdf]
