# Supplementary material for: Seroprevalence of Zika Virus in Wild African Green Monkeys and Baboons
Source: mSphere. 2017 Mar 8;2(2):e00392-16. doi: 10.1128/mSphere.00392-16 (PMC5343173; doi:10.1128/mSphere.00392-16)
Supplement: TABLE S2 [file sph002172248st2.pdf]

Non-human primate	Location name	GPS Coordinates	ΔOD[a]
Papio cynocephalus	Mikumi National Park, Tanzania	S7.1753 E37.4681	1.59
Papio cynocephalus	Mikumi National Park, Tanzania	S7.1753 E37.4681	1.527
Chlorocebus sabaeus	The Gambia	N13.6360 W14.9634	2.704
Chlorocebus sabaeus	The Gambia	N13.543611 W14.755556	2.003
Chlorocebus sabaeus	The Gambia	N13 23.340 W016 39.203	1.566
Chlorocebus sabaeus	The Gambia	N13.543611 W14.755556	2.667
